# Predicting Behavioral Target Mastery With Age, Intensity and Duration, Open Targets, and Maintenance Failure With Applied Behavior Analysis in Individuals With Autism

**DOI:** 10.7759/cureus.53964

**Published:** 2024-02-10

**Authors:** Tami Peterson, Jessica Dodson, Frederick Strale,

**Affiliations:** 1 Special Education, The Oxford Center, Brighton, USA; 2 Applied Behavior Analysis, The Oxford Center, Brighton, USA; 3 Biostatistics, The Oxford Center, Brighton, USA

**Keywords:** applied behavioral analysis (aba), maintenance failure, open targets, intensity and duration, age, autism spectrum disorder (asd), behavioral target mastery

## Abstract

Background

Researchers have studied the effects of age, treatment intensity, and treatment duration with applied behavior analysis (ABA) outcomes in autistic individuals. This study's primary objective was to evaluate the predictive capabilities of age, intensity, and duration of treatment, open behavioral targets, and behavior maintenance failure on behavioral target mastery.

Methods

A retrospective cohort of 100 autistic individuals treated with ABA with functional analysis and discrete trial training, mass trials, and naturalistic training were treated and analyzed. Target behavioral mastery data was collected via a retrospective chart review contained within the "Catalyst" tracking software. ABA treatment was administered for three months between March 19, 2023, and June 11, 2023. Multiple linear regression was performed using the percentage of behavioral targets mastered as the dependent variable. The independent variables were age, average trials to behavioral mastery, average teaching days to behavioral mastery, and percentage of behavioral targets that failed in maintenance.

Results

The multiple linear regression model was statistically significant (R=0.443, R²=0.196, adjusted R^2^=0.150, F(5, 87)=4.239, p=0.002). The average teaching days to mastery (β=0.416, p=0.019) and percentage of targets failed in maintenance (β=0.201, p=0.047) significantly predicted the percentage of behavioral targets mastered. However, age (β=0.079, p=0.419), average trials to mastery (β=-0.271, p=0.114), and open targets (β=0.184, p=0.081) did not significantly predict the percentage of behavioral targets mastered. The analysis of variance (ANOVA) resulted in non-significant (p>0.05) age group differences between the above variables.

Conclusions

The predictor variables average teaching days to mastery (intensity and duration) and percentage of targets failed in maintenance had a statistically significant effect on the percentage of behavioral targets mastered. The predictor variables age, average trials to behavioral mastery (intensity and duration), and open behavioral targets had a non-significant influence on the percentage of behavioral targets mastered. A non-significant difference between age groups was found in all study variables.

## Introduction

It is well documented that applied behavior analysis (ABA) is an effective intervention for children and adults with autistic spectrum disorder (ASD). Various researchers have studied the effects of age [1-4], treatment intensity [1,4-9], and treatment duration [4,10] on ABA treatment outcomes in children with autism.

Linstead et al. [11] commented that the effects of treatment duration require further study, citing Granpeesheh et al. [1] regarding their suggestion that using mastered learning objectives as a dependent variable in treatment studies would allow for the measurement of short-term outcomes which standardized diagnostic assessment scales are not designed to detect. Using mastered learning objectives as the dependent variable may be a strength because it provides a socially significant and general analysis of treatment progress. Linstead et al. [11] emphasized treatment-specific assessments of mastered learning objectives to measure short-term progress/failures as a notable outcome measure and the role of treatment duration and intensity as a significant predictor of behavioral target mastery.

Also, using large N designs to predict future learning rates and treatment-specific variables can provide clinicians, educators, policymakers, and parents insight into how children with ASD will respond to ABA treatment. Additional research on the relationship between treatment duration and skill acquisition may inform clinicians and parents regarding potential treatment outcomes, reduce attrition, and increase parental involvement in treatment [1].

The primary objective of this study is to evaluate the effectiveness of ABA treatment relative to the predictive capabilities of age, intensity and duration of treatment (as measured by average trials to behavioral mastery), intensity and duration of treatment (as measured by average teaching days to behavioral mastery), behavioral maintenance failure (as measured by the percentage of targets failed in behavioral maintenance), and open behavioral targets (behavioral targets that the individual has not mastered yet), on mastered learning objects as measured by the percentage of behavioral targets mastered.

The secondary objective of the study is to determine if an association exists between specific age group categories (1-4 years, 5-8 years, 9-12 years, 13-16 years, and 17-73 years) and intensity and duration of treatment (as measured by average trials to behavioral mastery), intensity and duration of treatment (as measured by average teaching days to behavioral mastery), behavioral maintenance failure (as measured by the percentage of behavioral targets failed in maintenance), and open behavioral targets (behavioral targets that the child has not mastered yet), on mastered learning objects as measured by the percentage of behavioral targets mastered.

## Materials and methods

Participants and setting

This study used a retrospective cohort of 100 total individuals (89 children, four adults, seven missing values for children's age) treated with ABA with functional analysis and discrete trial training, mass trials, and naturalistic training covering three months between March 19, 2023, and June 11, 2023. Target behavioral mastery data was collected via a retrospective chart review contained within the "Catalyst" tracking software. All autistic individuals were seen and treated at The Oxford Centers (TOC; Brighton and Troy, Michigan, United States), specializing in the mixed-methods approach to ABA utilizing discrete trial training, mass trials, and naturalistic environment training treatment modalities.

Before training, each individual received a treatment plan developed by one of the eight board-certified behavioral analysts (BCBAs) based on the individual's needs and goals. The individual is assigned to one of the 83 behavioral technicians and may have a 3-to-5 behavioral technician team over the three months. Appropriate materials are selected and set in rooms where individual discrete trial training and mass trials can occur or in a naturalistic setting where individuals can interact with others and experience functional and meaningful real-world situations. Each behavioral technician is assigned to a different individual daily, receiving, on average, 4-7 hours of treatment per day for a minimum of 25 hours a week.

Behavioral technician teams gathered specific behavioral and skill data relative to antecedent, behavior, and consequence of behavior, noting progress and fading prompts and reinforcements as the individual mastered the skills at 80% accuracy [12] and whether the individual was generalizing and maintaining the skill. Data was entered into a handheld "Catalyst" database and aggregated and updated daily into a central database.

Method of data collection and operational definitions of variables

Data was collected via retrospective chart review to gather data relative to autistic individuals treated with ABA. Manuscript generation and reporting adhered to Strengthening the Reporting of Observational Studies in Epidemiology (STROBE) guidelines. The dependent (predicted) variable was the percentage of behavioral targets mastered in the three months. The independent (predictor) variables were age in years, average trials to behavioral mastery (intensity and duration), average teaching days to behavioral mastery (intensity and duration), open behavioral targets, and percentage of behavioral targets failed in maintenance.

Dependent variable

The percentage of behavioral targets mastered in the three-month period measured the progress of an individual relative to the achievement of learning goals and/or skills. When the individual performed the task or skill correctly with 80% accuracy according to the criterion established by the BCBA, the individual was considered to have achieved mastery of that task or skill.

Independent variables

There were several independent variables in this study. First, age in years refers to chronological age according to a 365-day/52-week/12-month year. Second, average trials to behavioral mastery, a measure of treatment intensity and duration, refers to the number of behavioral responses required to achieve a level of behavioral performance (the mastery criterion) determined by the BCBA for a specific skill or behavior. Third, average teaching days to mastery, a measure of treatment intensity and duration, refers to how many days it took from when a target was first introduced to when it was mastered. Individuals receive five full days of ABA treatment per week using treatment protocols designed for each specific individual. Fourth, open behavioral targets refer to behaviors currently being taught and learned but have not been mastered yet. Fifth, the percentage of behavioral targets failed in maintenance refers to individual behaviors that have failed to be maintained over time. It means a failure to demonstrate or maintain a previously acquired skill or behavior after the required ABA antecedent has been withdrawn. Lastly, "Catalyst," an ABA data collection software, was used to generate automated progress reports for outcome data for discrete trial teaching targets with frequency and rate data. Mastery criteria for target behaviors are defined as the percentage of behavioral trials, minimum number of behavioral trials, and number of therapists above 80% criteria. Graphs in Catalyst are customized to track progress and/or lack of progress with targeted behaviors. Catalyst automatically determines mastered target behaviors as criteria are achieved.

Power analysis-study size

A retrospective power analysis was conducted using G*Power 3.1 [13] and indicated that a total sample size of n=29 participants would be required to demonstrate a high group effect size (0.80) for linear multiple regression: fixed model, with nominal alpha (α)=0.05 with a power equal to 0.955. Given multiple linear regression analysis parameters, there is a high likelihood that this current retrospective study with n=95 participants indicated an acceptable sample size criterion.

Statistical methods

IBM SPSS Statistics for Windows, Version 29.0 (Released 2022; IBM Corp., Armonk, New York, United States) was used for all descriptive and inferential analyses. The nominal α was set at 0.05. If p-values are less than 0.05 (p<0.05), a null hypothesis will be rejected, and statistical significance will be inferred. Descriptive demographics will be analyzed and presented, along with missing values. Summary statistics for the categorical variables, gender and race/ethnicity, and for the continuous variables, age, percentage of behavioral targets mastered, average trials to behavioral mastery, average teaching days to behavioral mastery, open behavioral targets, and percentage of behavioral targets failed in maintenance (mean and standard deviation, median, and range), will be generated.

A correlational analysis using Pearson r was performed on the variables percentage of behavioral targets mastered, age, average trials to behavioral mastery, average teaching days to behavioral mastery, open behavioral marks, and percentage of behavioral targets failed in maintenance to ascertain statistically or non-statistically significant relationships between variables.

A multiple linear regression analysis was performed using the percentage of behavioral targets mastered as the dependent (predicted) variable. The independent variables consisted of (1) age, (2) average trials to behavioral mastery, (3) average teaching days to behavioral mastery, (4) open behavioral targets, and (5) percentage of behavioral targets failed in maintenance.

A one-way analysis of variance (ANOVA) was performed to determine statistically significant differences between specific age group categories (1-4 years, 5-8 years, 9-12 years, 13-16 years, and 17-73 years) across the variables of (1) age, (2) average trials to behavioral mastery, (3) average teaching days to behavioral mastery, (4) open behavioral targets, (5) percentage of behavioral targets failed in maintenance, and (6) percentage of behavioral targets mastered. Corresponding boxplots were also produced and displayed.

Institutional Review Board (IRB)

This research study was conducted retrospectively from data obtained via chart review for clinical purposes. The study was submitted to the WIRB-Copernicus Group (WCG® IRB) for review and received an exemption (approval number: 1-11703366-1). The authors hereby certify that the analysis was performed in accordance with the ethical standards as put forth in the 1964 Declaration of Helsinki and its later amendments or comparable ethical standards. Please note that since obtaining the ClinicalTrials.gov Identifier NCT06043284, Oxford Recovery Center (ORC) has changed its name to The Oxford Center (TOC) (other study ID numbers: OxRS-01-2021).

## Results

Descriptive demographics

For the sample of 100 autistic individuals, the age in years was M=8.88 and SD=8.05, the median was 7, the minimum was 1, and the maximum was 73. There were seven missing values. There were 74 males (74%) and 25 females (25%). There was one missing value. There were 72 whites (72%), 12 Asians (12%), five American Indian/Alaska natives (5%), four Hispanics (4%), and seven unspecified (7%). With age categories, 18 children (18%) were in the 1-4-year category, 39 children (39%) were in the 5-8-year category, 20 children (20%) were in the 9-12-year category, 12 children (12%) were in the 13-16-year category, and four children (4%) were in the 17-73-year category. There were seven missing values. Please note that there were four subjects greater than 17 years old, i.e., 18 years old, 20 years old, 25 years old, and 73 years old.

Inferential statistics

A multiple linear regression analysis was performed on the dependent and independent variables, beginning with analyzing underlying assumptions. Before performing multiple regression procedures, assumption procedures indicated that this study achieved independence of observations, as the measurements for each autistic child were in no way influenced by or related to the measurements of other autistic children in the sample. Each child only counted as one observation. The value of one observation did not change or affect the value of another observation (no dependence). Bivariate scatterplots also determined a linear relationship between the dependent and independent variables. 

Homoscedasticity was confirmed with standardized residuals distributed evenly between -3 and +3. Minimal multicollinearity was detected as tolerance levels were >0.10 (0.305-0.971) and variance inflation factor (VIF) scores were <5 (1.03-3.28). However, a Pearson r=0.826, p<0.001 was detected in the correlation matrix between average trials to behavioral mastery and average teaching days to mastery variables. The normality of residuals was assessed via standardized residuals, which fell within an acceptable range (-1.724-1.968). Cook's distance found one significant outlier in the dependent variable, Cases 9, 29, and 34. Pearson correlations between the independent and dependent variables are presented in Table 1 below.

**Table 1:** Pearson correlations of dependent variable and independent variables *p<0.05, two-tailed; **p<0.01, two-tailed

		Percentage of behavioral targets mastered	Age in years	Average trials to behavioral mastery	Average teaching days to behavioral mastery	Open behavioral targets
Age in years	r	0.126				
	p-value (two-tailed)	0.228				
	N	93				
Average trials to mastery	r	0.142	-0.038			
	p-value (two-tailed)	0.159	0.716			
	N	100	93			
Average teaching days to mastery	r	0.281^**^	0.034	0.826^**^		
	p-value (two-tailed)	0.005	0.748	<0.001		
	N	100	93	100		
Open behavioral targets	r	0.254^*^	0.134	0.252^*^	0.359^**^	
	p-value (two-tailed)	0.011	0.199	0.011	<0.001	
	N	100	93	100	100	
Percentage of behavioral targets failed in maintenance	r	0.246^*^	-0.011	0.232^*^	0.198^*^	0.113
	p-value (two-tailed)	0.014	0.918	0.02	0.049	0.262
	N	100	93	100	100	100

Multiple regression

The overall multiple linear regression model was statistically significant (R=0.443, R²=0.196, adjusted R^2^=0.150, F(5, 87)=4.239, p=0.002). Ozili [14] put forth that an R^2^ between 0.10 and 0.50 (or between 10% and 50% when expressed in percentage) is credible in social science studies only when some or most of the predictor variables are statistically significant. Three out of the five predictor variables are statistically significant in this study. It was found that average teaching days to mastery significantly predicted the percentage of behavioral targets mastered (β=0.416, p=0.019). Also, the percentage of targets failed in maintenance significantly predicted the percentage of behavioral targets mastered (β=0.201, p=0.047). It was found that age did not significantly predict the percentage of behavioral targets mastered (β=0.079, p=0.419) and average trials to mastery did not significantly predict the percentage of behavioral targets mastered (β=-0.271, p=0.114) and open targets also did not significantly predict the percentage of behavioral targets mastered (β=0.184, p=0.081). See Table 2 below.

**Table 2:** Multiple linear regression coefficients Outcome variable: percentage of behavioral targets mastered; CI: confidence interval; VIF: variance inflation factor

		Unstandardized coefficients		Standardized coefficients	t	p-value	95% CI for B		Collinearity statistics	
		B	Std. error	Beta			Lower bound	Upper bound	Tolerance	VIF
	(Constant)	31.498	6.455		4.88	<0.001	18.668	44.328		
Predictor variable	Age in years	0.261	0.322	0.079	0.811	0.419	-0.379	0.901	0.971	1.03
Predictor variable	Average trials to mastery	-0.085	0.053	-0.271	-1.595	0.114	-0.19	0.021	0.319	3.13
Predictor variable	Average teaching days to mastery	1.099	0.46	0.416	2.391	0.019	0.185	2.013	0.305	3.279
Predictor variable	Open targets	0.268	0.152	0.184	1.765	0.081	-0.034	0.57	0.852	1.174
Predictor variable	Percentage of targets failed in maintenance	0.72	0.357	0.201	2.015	0.047	0.01	1.43	0.932	1.073

Results of the one-way ANOVA in Table 3 below indicated no statistically significant difference (p>0.05) across all age group categories on the percentage of behavioral targets mastered, average trials to behavioral mastery, average teaching days to behavioral mastery, open behavioral targets, and percentage of behavioral targets failed in maintenance.

**Table 3:** Association between specific age groups and target mastery: one-way ANOVA ANOVA: analysis of variance

		Sum of squares	df	Mean square	F	p-value
Average trials to mastery	Between groups	12904.17	4	3226.042	0.435	0.783
	Within groups	652152.7	88	7410.826		
	Total	665056.9	92			
Average teaching days to mastery	Between groups	284.263	4	71.066	0.693	0.599
	Within groups	9019.773	88	102.497		
	Total	9304.036	92			
Open targets	Between groups	2378.749	4	594.687	1.863	0.124
	Within groups	28088.82	88	319.191		
	Total	30467.57	92			
Percentage of targets mastered	Between groups	981.361	4	245.34	0.338	0.852
	Within groups	63889.07	88	726.012		
	Total	64870.43	92			
Percentage of targets failed in maintenance	Between groups	60.824	4	15.206	0.269	0.897
	Within groups	4976.73	88	56.554		
	Total	5037.555	92			

## Discussion

The current study was designed to evaluate the predictive capabilities of age, average trials to behavioral mastery (intensity and duration), average teaching days to behavioral mastery (intensity and duration), open behavioral targets, and percentage of behavioral targets that failed in mastery on behavioral target mastery. A noteworthy finding was that the predictor variables average teaching days to behavioral mastery (intensity and duration) and percentage of behavioral targets failed in maintenance had a statistically significant effect on the percentage of behavioral targets mastered. We also discovered that the variables age, average trials to behavioral mastery (intensity and duration), and open behavioral targets had a non-significant influence on the percentage of behavioral targets mastered.

In this study, one intriguing significant predictor variable of behavioral target mastery was the percentage of behavioral targets that failed in maintenance (β=0.201, p=0.047). It might be argued that this sample of autistic individuals possesses specific characteristics of what is referred to as "intellectual humility" [15] or a willingness to learn and progress, most likely prompted by the BCBA or behavioral technician.

Porter et al. [15] discussed the challenges and struggles inherent to learning. Individuals who embrace challenges and persist through difficulties tend to learn more and achieve higher levels of learning. Learners higher in intellectual humility behave in a mastery-oriented way: they take on challenges, exert tremendous effort, and persist despite setbacks. Boosting intellectual humility thus may offer a promising pathway to foster mastery behaviors and advance learning in autistic and normative individuals.

It is also noteworthy that the five variables, average trials to behavioral mastery (intensity and duration), average teaching days to behavioral mastery (intensity and duration), open behavioral targets, percentage of behavioral targets failed in maintenance, and percentage of behavioral targets mastered, analyzed in the one-way ANOVA with corresponding boxplots, displayed non-significant differences between specific age group categories (1-4 years, 5-8 years, 9-12 years, 13-16 years, and 17-73 years).

Virues-Ortega et al. [4] found that total intervention time, pre-intervention functioning, and age were the most prominent predictors of behavioral target accomplishment. Eldevik et al. [5] reported that the intervention intensity predicted IQ and adaptive behavior gains. Linstead et al. [11] discovered that treatment intensity and duration predicted mastery of behavioral learning objectives.

Comparable to Linstead et al. [11] and Granpeesheh et al. [1], our study found that the number of teaching days showed a statistically significant linear relationship with the number of mastered behavioral objectives. An increase in treatment hours increased the percentage of behavioral targets mastered. Similarly, Makrygianni and Reed [3] reviewed 14 studies and found that the effectiveness of behavioral programs for autistic children was significantly correlated with the intensity and duration of the programs.

However, unlike Granpeesheh et al. [16], we found no association between autistic individual's age and the percentage of behavioral targets mastered. Our one-way ANOVA indicated no significant differences (see Table 2 and Table 3) between individuals' age group categories. 

Limitations

Our study has many limitations, one of which is the general basic scope of the project based on the available data contained within Catalyst at TOC. This study did not take a detailed approach like Linstead et al. [11] where they analyzed the effects and impacts of intensity and duration of ABA intervention on learning across different treatment domains, including academic, adaptive, cognitive, executive function, language, motor, play, and social. This study did, however, follow the suggestions of Linstead et al. [11] and Granpeesheh et al. [1], in that we were able to use the number of mastered behavioral learning objectives determined based on direct observation as the dependent variable and variables including age, open behavioral targets, and behavioral targets failed in maintenance along with intensity and duration variables using a large N design [17,18].

Individuals with ASD typically receive several treatments, both traditional and complementary. Also, this study may not be representative of the entire ASD population, and it is difficult to detect differences between groups and to adjust for potential confounders fully. Comorbidities are common in individuals with autism. The heterogeneity of ASD implies the potential overlapping of symptoms of ASD and comorbidities. Also, the researchers in this study did not have data available relative to the numerous comorbidities that exist in autistic individuals.

## Conclusions

Researchers have commented that treatment intensity and duration effects require further study. They suggested that using mastered learning objectives as a dependent variable in treatment studies would allow for measuring short-term outcomes, which standardized diagnostic assessment scales are not designed to detect. A noteworthy finding in our study was that the predictor variables average teaching days to mastery (intensity and duration) and percentage of targets failed in maintenance had a statistically significant effect on the percentage of behavioral targets mastered. The variables age, average trials to mastery (intensity and duration), and open targets had a non-significant influence on the percentage of behavioral targets mastered. Five variables, average trials to mastery (intensity and duration), average teaching days to mastery (intensity and duration), open behavioral targets, percentage of behavioral targets failed in maintenance, and percentage of behavioral targets mastered, displayed non-significant differences between specific age group categories. One intriguing significant predictor of behavioral target mastery was the "percentage of targets failed in maintenance." It might be argued that this sample of autistic children possesses specific characteristics of what might be referred to as "intellectual humility," or a willingness to learn and progress, most likely prompted by the BCBA or behavioral technician.
